# Targeting the epigenetics of the DNA damage response in breast cancer

**DOI:** 10.1038/cddis.2016.85

**Published:** 2016-04-07

**Authors:** M F Montenegro, R González-Guerrero, L Sánchez-del-Campo, A Piñero-Madrona, J Cabezas-Herrera, J N Rodríguez-López

**Affiliations:** 1 Department of Biochemistry and Molecular Biology A, School of Biology, University of Murcia, Murcia 30100, Spain; 2 Nuffield Department of Clinical Medicine, Ludwig Institute for Cancer Research, University of Oxford, Headington, Oxford OX3 7DQ, UK; 3 Department of Surgery, University Hospital Virgen de la Arrixaca, Instituto Murciano de Investigación Biosanitaria, Murcia 30120, Spain; 4 Translational Cancer Research Group, University Hospital Virgen de la Arrixaca, Instituto Murciano de Investigación Biosanitaria, Murcia 30120, Spain

## Abstract

Cancer is as much an epigenetic disease as it is a genetic disease, and epigenetic alterations in cancer often serve as potent surrogates for genetic mutations. Because the epigenetic factors involved in the DNA damage response are regulated by multiple elements, therapies to target specific components of the epigenetic machinery can be inefficient. In contrast, therapies aimed at inhibiting the methionine cycle can indirectly inhibit both DNA and protein methylation, and the wide variety of genes and pathways that are affected by these methylations make this global strategy very attractive. In the present study, we propose an adjuvant therapy that targets the epigenetics of the DNA damage response in breast cancer cells and that results in efficient apoptosis and a reduction in distant metastases *in vivo*. We observed that a combined therapy designed to uncouple adenosine metabolism using dipyridamole in the presence of a new synthetic antifolate, 3-*O*-(3,4,5-trimethoxybenzoyl)-(−)-catechin, simultaneously and efficiently blocked both the folic cycle and the methionine cycle in breast cancer cells and sensitized these cells to radiotherapy. The treatment impeded the recruitment of 53BP1 and BRCA1 to the chromatin regions flanking DNA double-strand breaks and thereby avoided the DNA damage responses in breast cancer cells that were exposed to ionizing radiation. In addition, this hypomethylating therapy was also efficient in reducing the self-renewal capability of breast cancer-initiating cells and induced reversion of mesenchymal phenotypes in breast cancer cells.

Radiation is an important therapeutic intervention in many tumor types, and it has been utilized to treat different type of cancers as either a primary therapy or an adjuvant treatment. However, local recurrence after radiation continues to be a major hindrance to using this type of therapy. For example, breast cancer (BC) cells are relatively refractory to ionizing radiation (IR)-induced DNA damage and apoptosis.^1^ Combinations that use radiation with biological modifiers constitute a main research objective for oncologist,^2^ and it is widely accepted that blocking DNA repair mechanisms improves radiation therapy in refractory BCs, especially in those with p53 mutations that exhibit increased resistance to apoptosis. Thus, patients with p53-mutated tumors represent a category of patients who might benefit from the combined use of radio- and chemotherapy.

Because histone post-translational modifications influence the structure and functions of chromatin, histone lysine methylation may control many fundamental biological processes.^3^ For instance, the dimethylation of histones seems to have an important role in the DNA damage response (DDR).^4, 5^ The checkpoint mediator 53BP1 is directly recruited to the chromatin regions flanking DNA double-strand breaks (DSBs),^6, 7^ and in mammals, this occurs via an interaction with histone H4, which is dimethylated at Lys20, or histone H3, which is dimethylated at Lys79. MMSET (also known as NSD2 or WHSC1) and DOT1L, respectively, have been implicated as the methylases involved in these processes.^4, 5^ Because 53BP1 is a critical regulator of DDR signaling, knockdown of MMSET or DOT1L resulted in a reduction in IR-induced 53BP1 focus formation, defects in DNA repair and increased sensitivity to IR.^4, 5^

In addition to 53BP1, BRCA1 also has a decisive role in DNA DSB repair mechanisms.^8^ Whereas 53BP1 inhibits end resection and facilitates non-homologous end-joining primarily during the G1 phase of the cell cycle, BRCA1 promotes DNA end resection and homologous recombination during the S/G2 phases. This competitive relationship is critical for genome integrity during cell divisions. Recent investigations have indicated that BRCA1 activity may be regulated by protein methylation.^9, 10^ Lee *et al.*^9^ found that p300 methylation by coactivator-associated arginine methyltransferase 1 (CARM1) may be an important mechanism that regulates the activity of BRCA1 because the recruitment of BRCA1 to the p53-binding region of the p21 promoter in response to DNA damage required the methylation of Arg754 of p300 by CARM1. It was also demonstrated that the direct methylation of BRCA1 by protein arginine methyltransferase 1 (PRMT1) influenced its transcriptional cofactor functions.^10^ These results suggest that methylation may influence either the ability of BRCA1 to bind to specific promoters or its protein–protein interactions, which can alter the recruitment of BRCA1 to these promoters. These data indicate that alterations in the levels of cellular methylation can modify BRCA1-governed pathways in BC cells.

On the basis of these observations, we designed a strategy to prevent DNA damage repair in BC cells using a hypomethylating therapy (HMT; Figure 1) as an adjuvant to radiotherapy. Although the specific inhibition of DNA or protein methylases primarily resulted in specific unmethylated products, therapies based on inhibiting the methionine cycle result in the indirect inhibition of both DNA and protein methylation.^11, 12^ The wide variety of genes and pathways that are affected by DNA and protein methylation make this global strategy very attractive. The effects of a HMT would be reflected in the sum of its multiple effects on cellular physiology, and it is likely that the net effect of a HMT would be therapeutically favorable. Indeed, this nonspecificity can be viewed as advantageous because multiple defects are corrected simultaneously. Therefore, it may be possible that the application of new therapies to target the epigenetic machinery of cancer cells could be used to switch resistant cancer cells to more sensible phenotypes, to promote E2F1-dependent apoptosis in p53-defiant tumors, to reduce cancer-initiating cells (CICs), to prevent metastatic pathway activities and/or to sensitize tumor cells to radiotherapy.^11^

## Results

### HMT efficiently inhibits H4K20 and H3K79 dimethylation and suppresses DNA-damage-induced focus formation of 53BP1 in BC cells

An increase in total H4K20me2 levels was found using western blot analysis after IR; however, it was only observed at high doses of radiation.^4^ It is possible that, although local increases in H4K20me2 at DBSs were induced by low doses of IR, they were masked from detection by western blot analysis as a result of the high basal levels of H4K20me2 that occurred throughout the genome.^4^ Therefore, to visualize changes in H4K20me2 levels in BC cells, we used high doses (120 Gy) of IR (Figure 2a; Supplementary Figure S1). As shown in these figures, IR significantly increased dimethylation at H4K20, the levels of which were dramatically reduced after pretreatment of the cells with the HMT. Because MMSET, the methylase responsible for H4K20 dimethylation, requires the presence of phospho-H2AX (γH2AX) for its recruitment to DSBs, we next examined the effect of this HMT on the level and IR-dependent induction of phosphorylated H2AX (Figure 2a). As expected, IR strongly increased the phosphorylation of H2AX in MDA-MB-231 cells; however, no differences were observed when the cells were irradiated after treatment with the HMT (IR/HMT; Figure 2a). Because the HMT affected neither the interaction between MMSET and γH2AX nor MMSET focus formation after the cells were exposed to IR (Figure 2b), these results appeared to indicate that the HMT efficiently inhibited the catalytic function of MMSET in BC cells.

Hypomethylating conditions are thought to inhibit cellular SAM-dependent methylases, and no specificity was demonstrated in the HMT after H3K79me2 levels were evaluated in BC cells (Figure 2a; Supplementary Figure S1b). Although H3K79 dimethylation slightly increased after IR, the HMT was able to significantly reduce this methylation, presumably via the indirect inhibition of DOT1L methyltransferase. Dimethylation of both H4K20 and H3K79 has an important and direct role in the DDR because it has been demonstrated to modulate the recruitment of 53BP1 and its focus formation.^4, 5^ Indeed, the HMT-induced decreases in H4K20 and H3K79 dimethylation were accompanied by a significant decrease in the IR-damage-induced focus formation of 53BP1, but not of γH2AX, in BC cells (Figure 2c). Because 53BP1 is a critical regulator of signaling DNA damage and repair at sites of DNA damage induced by IR, these results predict deleterious effects on cells treated with this type of adjuvant therapy.

### Hypomethylation of the BRCA1 protein impedes its nuclear translocation upon exposure to IR

Because the functions of BRCA1 have been shown to be controlled by protein methylation,^10^ we next sought to determine whether the HMT modulated the methylated status of this protein. For these assays, whole-cell extracts from control and HMT-treated cells (MDA-MB-231 and MCF7) were immunoprecipitated with anti-BRCA1 or anti-IgG antibodies. As previously demonstrated,^10^ immunoblotting of electrophoresed proteins with an anti-R-methyl antibody showed that BRCA1 was methylated at arginine residues in both MDA-MB-231 and MCF7 cells (Figure 3a). Consistent with its proposed demethylating activity, we observed that the HMT caused a strong reduction in BRCA1 methylation at arginine residues in both cell lines (Figure 3a). Recently, it was suggested that the methylation status of the BRCA1 protein may have an important role in its cellular localization.^10^ However, whether the methylation status of BRCA1 affects its functions under conditions involving genotoxic stress has not been investigated. To answer this question, we labeled BRCA1 in BC cells using an anti-BRCA1 antibody, and its localization was then analyzed using confocal microscopy (Figures 3b and c). Resting MCF7 and MDA-MB-231 cells demonstrated some differences in BRCA1 localization, with BRCA1 being exclusively localized in the cytosol of untreated MCF7 cells. However, the response to IR was similar in both cell lines; thus, after IR, cytosolic BRCA1 was clearly transported into the nucleus, and at 1 h after irradiation, BRCA1 was mainly localized at nuclear foci. Interestingly, epigenetic intervention, using the HMT immediately before IR, not only increased the cytosolic localization of BRCA1 but also suppressed DNA-damage-induced focus formation of BRCA1 in BC cells (Figures 3b and c). Next, we silenced PRMT1 in MCF7 cells to determine whether the methylation state of BRCA1 was implicated in its differential localization. Knockdown of PRMT1 resulted in the cytosolic accumulation of BRCA1 after IR and in an increase in susceptibility in MCF7 cells to IR-induced apoptosis (Figure 3d). Because BRCA1 needs to be translocated into the nucleus to perform its DNA-repairing functions,^13^ these results indicate that hypomethylating conditions may prevent BRCA1-dependent DNA repair in BC cells that are subjected to IR.

### The HMT inhibits cell cycle arrest and impedes DNA repair in irradiated BC cells

Cells exposed to IR subsequently arrest in various phases of the cell cycle to repair damaged DNA, and, depending on the extent of the damage, such cells can either enter mitosis or die via apoptosis.^14^ Therefore, we analyzed whether the HMT inhibited IR-induced cell cycle arrest in MCF7 and MDA-MB-231 cells (Figure 4a). Although cells have been observed to accumulate in the G2/M phase of the cell cycle in the MCF7 and MDA-MB-231 cell lines,^15, 16^ we observed that arrest in this phase of the cycle was increased in MDA-MB-231 cells at 24 h post IR (Figure 4a). However, MCF7 cells exposed to the same experimental conditions were arrested in both the G0/G1 and G2/M phases (Figure 4a). Treatment of these cell lines with the HMT before IR restored the proportion of cells in S-phase cell and differentially modulated the sub-G0/G1 and arrested populations of cells in MCF7 and MDA-MB-231 cells.

These data indicate that the induced epigenetic intervention rescued discrete BC cell populations from cell cycle arrest and impeded DNA repair. To confirm these effects, we next investigated repair at clustered lesions within DNA/chromatin by analyzing focus formation and the persistence of foci of *γ*H2AX in irradiated MDA-MB-231 cells and in cells subjected to a double treatment regimen (IR/HMT). A time course for γH2AX focus formation in MDA-MB-231 cells was developed using immunocytochemistry, and it indicated that the activation of this protein peaked at ~1 h after IR (Figure 4b). Thereafter, at 24 h post irradiation, the amount of γH2AX was completely reduced in cells subjected to only IR. Because γH2AX is a sensitive indicator of both DNA damage and DNA replication stress, these data indicate that the MDA-MB-231 cells were able to completely repair their DNA after IR stress. However, when IR was administered in the presence of the HMT, the cells were not able to repair their DNA, as indicated by the continuous activation of γH2AX in nuclear foci (Figure 4b). When we used assays to investigate the integrity of DNA in MCF7 and 4T1 cells, we observed a high level of DNA fragmentation in the cells that were treated with IR/HMT. However, this DNA-damaged effect was not observed in the cells treated with IR (Figure 4c).

### The HMT sensitizes BC cells to IR-induced apoptosis

MCF7 breast carcinoma cells are representative of cancer cells that do not readily undergo apoptosis.^17^ Although they harbor a functional *p53* gene, MCF7 cells are especially resistant to IR-induced apoptosis. Instead, it has been observed that IR induces premature senescence in this cell line.^18^ We performed apoptosis and senescence assays, and confirmed both responses in MCF7 cells that were exposed to IR: resistance to apoptosis and susceptibility to senescence (Figure 4d). As shown in this figure, a large population of MCF7 cells became senescent after IR; however, when IR was combined with the HMT, we found that radiation was able to induce apoptosis in a large fraction of the cells.

Other BC cell lines harboring p53 mutations, such as MDA-MB-231 and 4T1, are highly resistant to both apoptosis and the induction of senescence after IR. Although we recently demonstrated that the HMT highly sensitized these cells to E2F1-dependent apoptosis,^12, 19^ in this study, we observed a strong synergy between this treatment and radiotherapy (Figure 4e). As shown in Figure 4d, radiation of MDA-MB-231 and 4T1 cells in the presence of HMT significantly increased the proportion of apoptotic cells with respect to HMT-treated cells. Finally, to compare a global hypomethylating strategy to other strategies used to target specific components of the epigenetic machinery in cancer cells, we evaluated the effect of combining IR with either 5-Aza-dC, a DNA-hypomethylating agent, or (R)-PFI-2, a potent and selective inhibitor of SET9.^20^ As shown in Figure 4d, these combined treatments were unable to induce apoptosis in these BC cells.

### Hypomethylating conditions affect stem cell and mesenchymal phenotypes in BC cells

Breast CICs are functionally defined by their ability to form mammospheres from a single cell *in vitro*. To examine the impact of the HMT in combination with fractionated IR on MCF7-derived mammospheres, we plated primary mammospheres at single-cell densities and evaluated secondary mammosphere formation 7 days later. As has been previously shown,^21^ fractionated radiation increased the rate of self-renewal in CICs, whereas treatment with HMT (either with or without IR) significantly decreased self-renewal in these cells (Figure 5a). In addition, IR induced stemness in MCF7 cancer cells (Figure 5b), and we also observed that irradiation accelerated dedifferentiation and cellular dissociation in primary mammospheres (Figure 5c).^22^ The observed reduction in cell–cell adhesion in these dedifferentiated mammospheres was paralleled by a marked decrease in E-cadherin expression and a gain in SOX2 (Sex-determining region Y-Box2), a key factor that is capable of inducing pluripotency in somatic cells.^23^ However, primary mammosphere cultures irradiated in the presence of the HMT continued to maintain a more differentiated phenotype and to demonstrate reduced expression levels of SOX2 (Figure 5c).

Another characteristic of CICs is their ability to acquire a mesenchymal phenotype.^24^ Diverse lines of evidence have suggested a possible link between less differentiated stem cells and the mesenchymal-like cells that are generated by EMT.^24^ In this study, we observed that the HMT reversed the fibroblastic mesenchymal morphology of MDA-MB-231 cells by upregulating epithelial markers, such as E-cadherin, and by downregulating mesenchymal markers, such as vimentin and N-cadherin (Figure 5d). In agreement with these expression patterns, the HMT greatly inhibited the ability of MDA-MB-231 cells to migrate (Figure 5e), which indicates that this modality of adjuvant therapy may also reduce tumor metastasis in BCs.

### The HMT highly sensitizes BC tumors to radiotherapy *in vivo*

To determine the antitumorigenic and antimetastatic efficacy of this therapeutic application *in vivo,* we used mouse 4T1 cells expressing a luciferase reporter as a BC model. Compared with untreated mice, radiotherapy (at a total of 30 Gy) or HMT alone did not significantly reduce tumor growth. However, the combination IR/HMT therapy was highly efficient in reducing tumor areas and inducing apoptosis in solid tumors as determined by a DNA fragmentation assay (Figures 6a and b). Mice treated with this combination of radio- and chemotherapy had better survival rates (Figure 6c), which, together with our previous results showing that the HMT reversed the mesenchymal phenotype of BC cells and repressed the mammosphere-forming capacity of these cells after radiation, also indicates that this treatment may be effective in reducing distant metastasis. To explore this possibility, luciferase-tagged 4T1 cells were injected into the mammary path of Balb/c mice, and tumor expansion was measured after treatments were applied. Luciferase imaging showed that 87% of the control mice presented distant metastases that were localized mainly in the lungs, the bones and/or different mammary paths that were displaced from the location at which the tumor was injected (Figure 6d). Treatment of animals with a radiotherapy regimen did not reduce the formation of distant metastases. In contrast, and compared with the untreated mice, a nonsignificant increase was observed in the number of mice with metastases (Figure 6c). Whether this nonsignificant increase in the number of mice with metastases was related to resistance or to the gain in aggressiveness of tumor CICs after fractionated radiation^21^ is a question that was not answered. Importantly, treating mice with the HMT reduced the number of mice with distant metastases compared with the control groups; however, a stronger reduction was observed when animals were treated with a combination of HMT and radiotherapy (Figure 6c).

## Discussion

**Figure 1:**
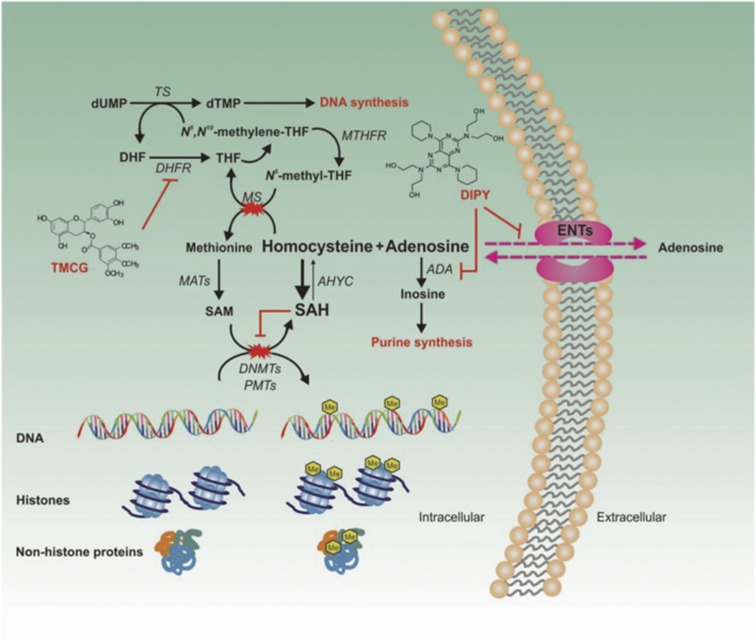
The strategy used to target the epigenetic machinery of BC cells. Experimental results, accumulated during the last decade, have suggested that the methionine cycle may be a valuable therapeutic target.^40, 41^ In a recent study, we observed that a combined therapy aimed at uncoupling adenosine metabolism using dipyridamole (DIPY) in the presence of a new synthetic antifolate (TMCG) simultaneously and efficiently blocked both the folic and methionine cycles in BC cells, resulting in massive cell death.^12^ The TMCG/DIPY combination acted as a HMT that reactivated RASSF1A expression and induced E2F1-mediated apoptosis in BC cells. The schematic illustrates the methionine cycle and its connections with several metabolic and survival cell pathways. Adenosine is efficiently metabolized by specific enzymes (such as ADA and adenosine kinase) before its use in purine nucleotide synthesis, which is particularly important for DNA synthesis in highly proliferating cells. Excess adenosine can be transported out of the cells by ENTs, which are bidirectional transporters that allow adenosine release and uptake by facilitating diffusion along its concentration gradient. However, in the presence of an antifolate compound, adenosine accumulation may become a severe problem for the cell. In folate-deficient cells, the resulting accumulation of homocysteine drives AHYC to catalyze the energetically favorable reverse reaction and to synthesize SAH, a potent inhibitor of cellular methyltransferases. ADA, adenosine deaminase; AHYC, S-adenosylhomocysteine hydrolase; DHF, dihydrofolate; DHFR, dihydrofolate reductase; DNMT, DNA methyltransferase; dTMP, deoxythymidine 5′-monophosphate; dUMP, deoxyuridine monophosphate; ENT, equilibrative nucleoside transporter; MAT, methionine adenosyltransferase; MS, methionine synthase; MTHFR, 5,10-methylenetetrahydrofolate reductase; PMT, protein methyltransferase; SAH, S-adenosylhomocysteine; SAM, S-adenosylmethionine; THF, tetrahydrofolate; TS, thymine synthase

**Figure 2:**
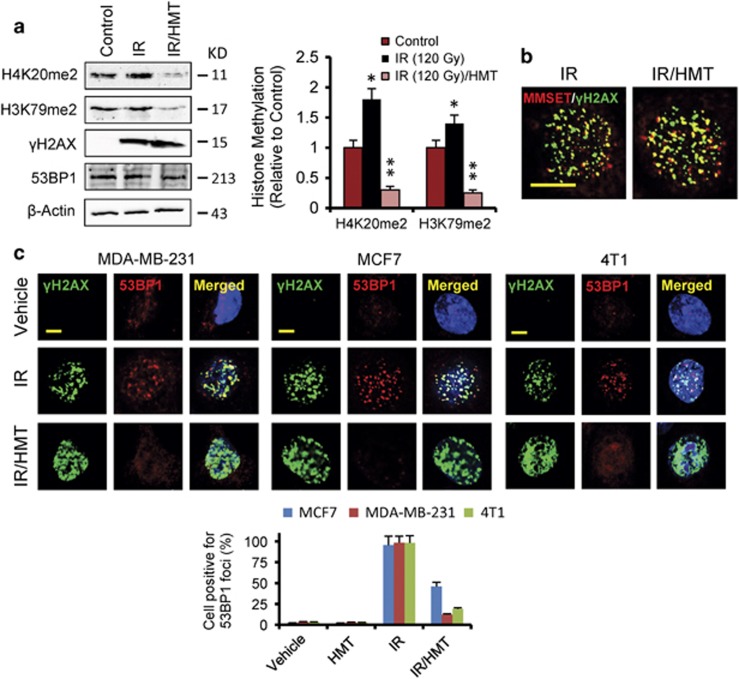
The HMT suppresses DNA damage-induced focus formation of 53BP1 in BC cells. (**a**) The total levels of H3K79me2, H4K20me2, γH2AX and 53BP1 were examined in MDA-MB-231 cells using western blot analysis following the indicated treatments. At 1 h after irradiation (120 Gy), the cells were collected and analyzed. Histone methylation (histogram) was estimated by integrated optical density (IOD) in western blots after normalization to the *β*-actin IOD. The values represent the mean from two experiments performed in triplicate. *P<0.05 when compared with control experiments and **P<0.05 when compared with both control and IR experiments. (**b**) The association between MMSET and γH2AX was assessed using immunofluorescence staining. MDA-MB-231 cells were irradiated (5 Gy), and, 1 h later, the cells were stained using the indicated antibodies. Scale bar, 10 *μ*m. (**c**) Immunofluorescence staining of specified BC cells was performed after the indicated treatments. The cells were irradiated (5 Gy), and, 1 h later, the cells were stained using the indicated antibodies. Scale bar, 5 *μ*m. The histogram demonstrates the quantification of the cells that were positive for 53BP1 foci, which were statistically significantly reduced (*P*<0.05) when cells were irradiated in the presence of HMT compared with the cells subjected to only IR

**Figure 3:**
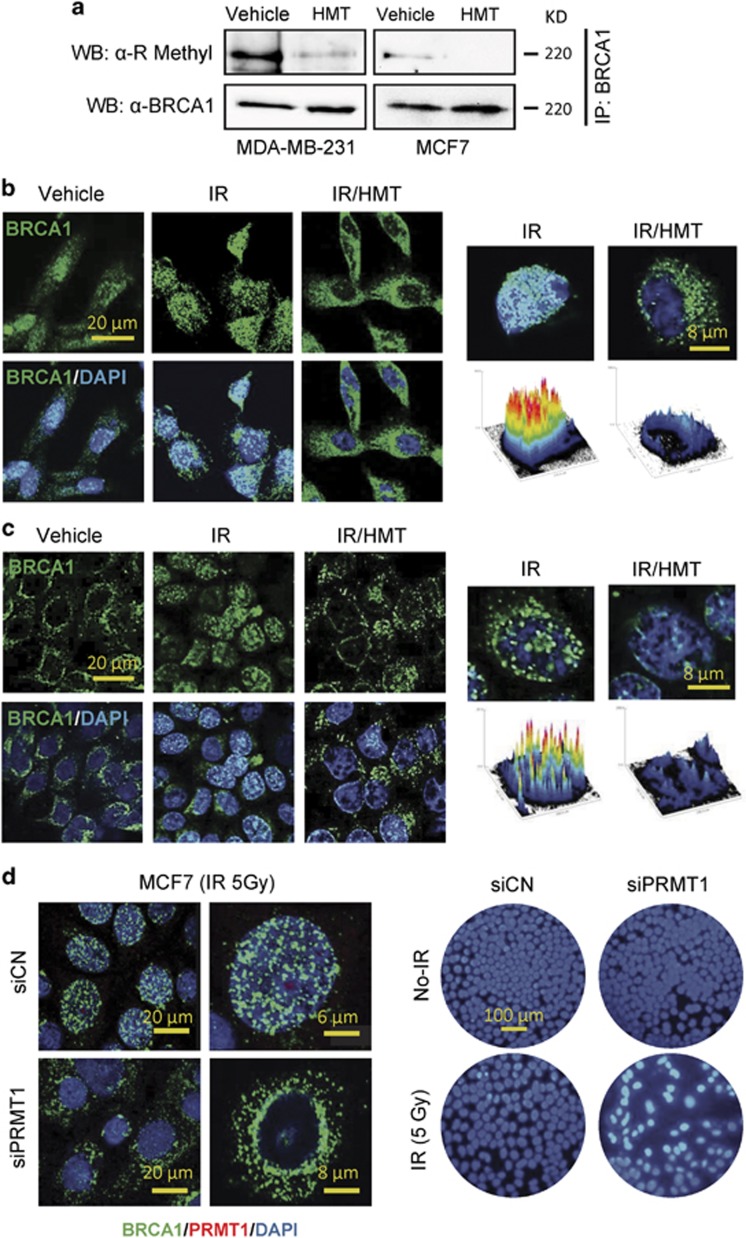
The methylation status of BRCA1 alters its cellular localization. (**a**) MDA-MB-231 and MCF7 cells were treated with the HMT to analyze the effect of inhibiting BRCA1 methylation following treatment. Two milligrams of whole-cell protein extracts were immunoprecipitated using an anti-BRCA1 antibody, separated using SDS-PAGE and probed using anti-R-methyl and anti-BRCA1 antibodies. The negative controls were immunoprecipitated using an anti-IgG antibody, which did not result in representative bands when similar blotting conditions were used (data not shown). The results are representative of three independent experiments. The localization of BRCA1 in MDA-MB-231 (**b**) and MCF7 (**c**) cells after the indicated treatments. The cells were irradiated (5 Gy), and, 5 h later, the cells were stained using the indicated antibodies. Histograms are shown to represent the intensity of BRCA1 staining in the nucleus. These results were quantified using the ImageJ-NIH software (Bethesda, MD, USA). (**d**) The effect of IR (5 Gy) on the localization of BRCA1 and apoptosis in PRMT1-depleted MCF7 cells. An analysis of BRCA1 was performed using confocal microscopy at 5 h post irradiation (left panel), and assays to analyze apoptosis were performed at 48 h after IR using Hoechst staining (right panel). Strong nuclear staining after IR was observed only in the siPRMT1 cells

**Figure 4:**
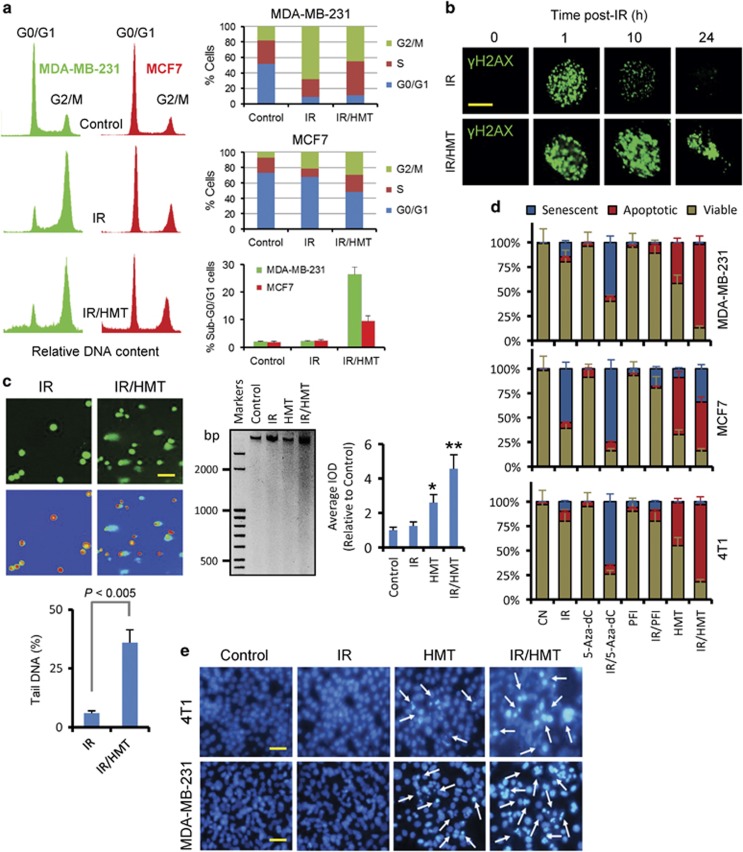
The HMT impeded DNA damage repair and sensitized BC cells to IR-induced apoptosis. (**a**) Cell cycle assays were performed using flow cytometry of MDA-MB-231 and MCF7 cells following the indicated treatments. When irradiated (5 Gy), cell cycle profiles (left panels) were obtained and analyses (right panels) were performed at 24 h post irradiation. Assays were performed in triplicate, and differences in the cell cycle populations were found to be statistically significant (*P*<0.05) when treated cells were compared with control cells (**b**). Nuclear focus formation of γH2AX was observed following double-stranded DNA damage. Scale bar represents 8 *μ*m. (**c**) Left panels, comet assay of MCF7 cells that were treated with IR or IR/HMT. Pictures were obtained at 24 h after IR (5 Gy). Hydrogen peroxide was used as a positive control (data not shown). The scale bar (100 *μ*m) refers to both panels. Right panel, DNA ladders of treated 4T1 cells 24 h after IR (10 Gy). Equal volume of lysate was loaded in all lanes and electrophoresis was performed on 2% agarose gel. The average IOD calculated by densitometry between 3000 and 450 bp was used to quantify DNA ladders (histogram). *P<0.05 when compared with control and IR-treated cells; **P<0.05 when compared with other treatments. (**d**) Responses in BC cell lines to the indicated treatments. At the zero time, the cells were treated with the HMT, 5-Aza-dC (1 *μ*M) or (R)-PFI-2 (20 *μ*M). On day 3, the cells were irradiated (5 Gy), and apoptosis or senescence was assayed on day 6. When the cells were not irradiated, the analyses of apoptosis or senescence were performed on the sixth day. (**e**) The images show the effect of the HMT, IR and IR/HMT treatments on apoptosis in MDA-MB-231 and 4T1 cells, as determined using fluorescence microscopy after staining the DNA using Hoechst 33342. Analyses of apoptosis were performed at 72 h after IR (5 Gy). The scale bar (100 *μ*m) refers to both panels

**Figure 5:**
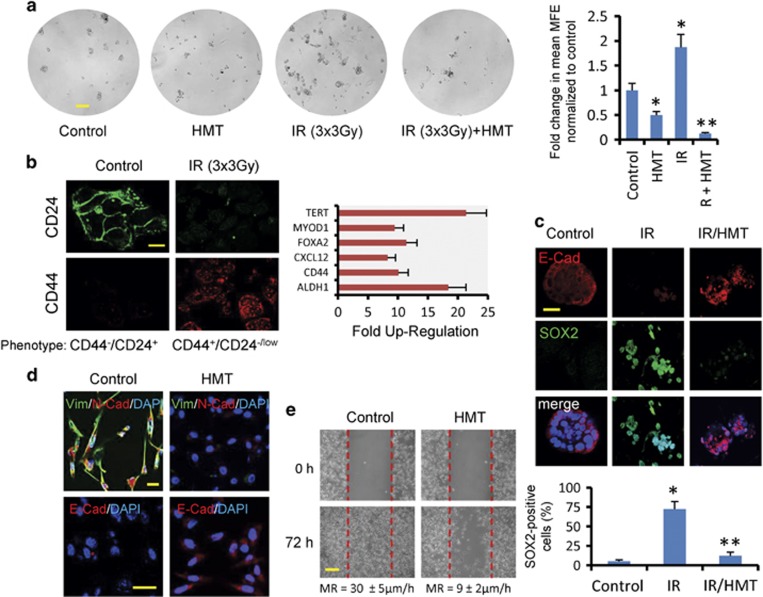
(**a**) The effects of fractionated radiation on secondary mammosphere-forming efficiency (MFE) in MCF7 cells. Primary MCF7 mammospheres were plated at single-cell densities and irradiated (3 Gy on three consecutive days) in the absence or presence of the HMT. Four days later, MFE was calculated. Scale bar refers 100 *μ*m. *P<0.05 compared with the control mammospheres. **P<0.05 compared with the irradiated mammospheres. (**b**) IR induces stemness in survivor cells. MCF cells were irradiated (5 Gy), and, after 72 h, the surviving cells were cultured in adherent plates. At 5 days after replating, the cells were analyzed using immunofluorescence for CD24 and CD44, and the expression levels of 84 genes related to the identification, growth and differentiation of stem cells were analyzed using PCR arrays. In comparison with resting MCF7 cells, IR-survivor cells showed elevated levels of some recognized stemness markers (*P*<0.001). Scale bar refers 40 *μ*m. (**c**) IR at 10 Gy accelerated dedifferentiation in MCF7-derived mammospheres. After 3 days, the morphological changes in the IR-treated primary mammospheres were paralleled by a decrease in E-cadherin (red) and the enrichment of SOX2 (green), as determined using confocal microscopy. However, the HMT inhibited the dedifferentiation of IR-treated primary mammospheres. Scale bars refers 20 *μ*m. The number of SOX2-positive cells was expressed as a percentage after 200 cells were counted in different microscopic fields. The bars indicated±S.D. from three independent experiments. *P<0.05 compared with the control mammospheres. **P<0.05 compared with both the control and the irradiated mammospheres. (**d**) The expression levels of vimentin (green), N-cadherin (red) and E-cadherin (red) were analyzed using immunofluorescence staining in MDA-MB-231 cells before and after 72 h of treatment with the HMT. Scale bar refers to 20 *μ*m. (**e**) Migration was analyzed in the control MDA-MB-231 cells and the cells treated with the HMT using wound-healing assays. A representative experiment is shown, and the statistical analysis for the migration rate (MR) is described (mean±S.D. from five independent experiments; *P*<0.002). Scale bar refers to 100 *μ*m

**Figure 6:**
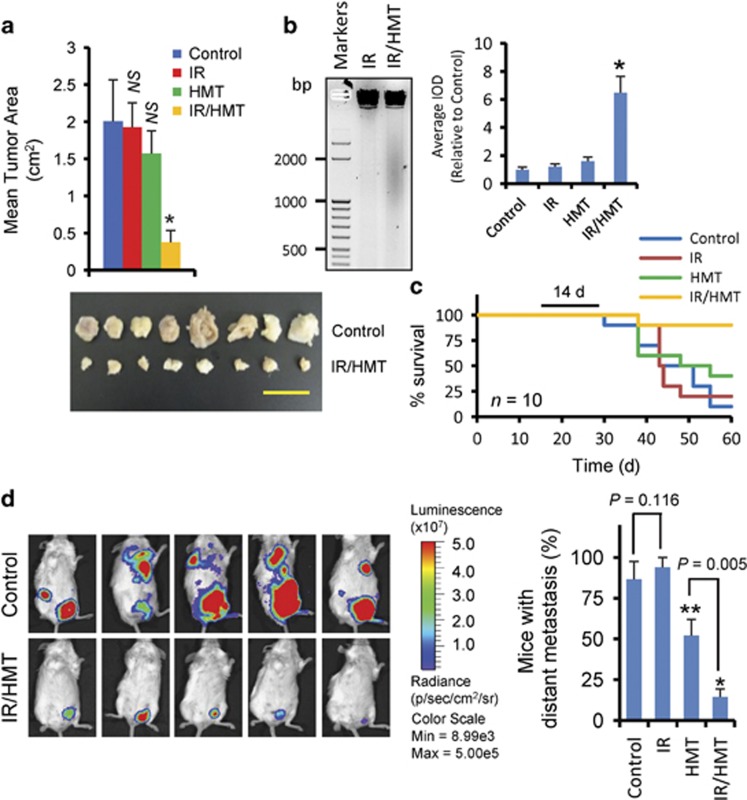
The HMT sensitized BC cells to radiotherapy *in vivo*. (**a**) The mean tumor size in the BALB/c mice bearing 4T1 BCs at 40 days post injection (*n*≥8 for each model). Differences between the IR/HMT-treated mice and the mice in the other indicated groups were found to be statistically significant (*P<0.001). NS, not statistically significant compared with the control. The image shows the tumor sizes at 40 days after the tumor cells were injected into the control and IR/HMT-treated mice. Scale bar refers to 1 cm. (**b**) Apoptosis in 4T1-induced tumors was determined using a DNA ladder assay. DNA from frozen tumors was isolated and analyzed with electrophoresis on 2% agarose gel. IOD quantification was performed as specified in Figure 4c. *P<0.05 when compared with other treatments. (**c**) Survival after 14 days of treatment (black line) in the BC mouse models was examined (*n*=10 for each model). (**d**) Luciferase imaging of the vehicle (control) and IR/HMT-treated mice at 40 days post-tumor cell injection. Firefly luciferin (120 mg/kg of mouse) was injected intraperitoneally. The values are representative of three independent experiments. A histogram representing the percentage of mice with distant metastases at 40 days post-tumor cell injection (*n*≥7 for each model). Metastases were determined by identifying cells expressing luciferase in organs other than the organs that were initially injected, and the values (means±S.D.) shown are representative of three independent experiments. *P<0.001 and **P=0.024 compared with the vehicle-treated mice

Epigenetics and metabolism are highly and reciprocally interconnected.^25, 26^ In the present study, we have proposed an adjuvant therapy that targets the epigenetics of the DDR in BC cells and resulted in efficient apoptosis and the reduction of distant metastases *in vivo*. The wide variety of methyltransferases that are affected by this hypomethylating treatment strongly suggests that it disrupts the epigenetic machinery of BC cells and that its effects are mainly associated with a deficit in SAM in treated cells (Figure 1). Another important observation from this study is that this combined strategy was active on BC cells independently of their ER/PR status and, therefore, it may be useful for the treatment of different molecular and histological subtypes of BC.

Here, we observed that this HMT impedes the recruitment of 53BP1 and BRCA to the chromatin regions flanking DSBs, thereby inhibiting DDR signals in BC cells treated with IR. Although inhibition of H4K20 and H3K79 dimethylation clearly explains the decrease observed in IR damage-induced focus formation in 53BP1,^4, 5^ the effect of hypomethylating conditions on the localization of BRCA1 in BC cells after IR was less clear. BRCA1 localization has an important role in the functions of this protein, which has both cytoplasmic and nuclear targets. BRCA1 has been found to possess two nuclear localization signals (NLSs) and one nuclear export sequence that guide the shuttling of BRCA1.^27^ However, the mechanisms underlying this shuttling process are not clearly understood. Interestingly, both NLSs are located in the vicinity of the BRCA1 region that has been identified to be methylated by PRMT1.^10^ Specifically, NLS1 is located at residues 501–507, and NLS2 is located at residues 606–615. Furthermore, the phosphorylation of T508 at the Akt consensus phosphorylation motif immediately adjacent to NLS1 resulted in cytoplasmic accumulation of BRCA1,^28^ suggesting that methylation of this region might be involved in the regulation of the shuttling mechanisms of BRCA1.

The identification and characterization of CICs enriched in stem cell-like functions and the establishment of a link between CICs and therapy resistance have been main focuses of cancer research in recent years.^29^ Later studies have suggested several interconnected mechanisms to explain CIC enrichment after radiation, and all of them appear to be related to tumor heterogeneity.^30, 31^ Thus, it has been suggested that the enrichment in CICs observed in tumors that have been subjected to radiation therapy may result, in part, from the delivery of sublethal doses of the treatment and the efficient radical scavenging system in the CICs.^32^ Sublethal doses of radiation are sufficient to induce senescence in non-CICs, to force CICs to acquire several new properties that are related to cell cycle progression, and to maintain or enhance stem cell characteristics in pretreated CICs. Moreover, it has been shown that during tumor metastasis, which is often enabled by EMT,^33^ disseminated cancer cells appear to require the ability to self-renew to spawn macroscopic metastases, similar to stem cells. This raises the possibility that the EMT process, which enables cancer cell dissemination, may also impart self-renewal capabilities to disseminating cancer cells.^34^ Importantly, the therapy proposed in this study efficiently repressed mammosphere formation after fractionated radiation and was able to revert mesenchymal phenotypes in BC cells. Increasing our understanding of the mechanisms by which the inhibition of the epigenetic machinery of BC cells can reduce stem cell activity in breast tumors and eliminate their resistance to radiation is an important issue that deserves further investigation. At this respect, it has been recently reported that therapeutic inhibition of the constitutive activation of poly-ADP-ribose polymerase (PARP) compromises stem cell phenotypes and survival in glioblastoma-initiating cells.^32^ Interestingly, inhibiting PARP1 enhanced the cytotoxicity of DNA-damaging agents in cancer cells, and, more recently, it was shown that the activity of this enzyme was enhanced after its SMYD2-dependent methylation at Lys528,^35^ suggesting a functional link between the methylation status of CICs and their capacity to resist sublethal doses of radiation.

The resistance of cancers to general chemotherapeutics, their evasion of cellular suicide and their resistance to apoptosis are primarily related to the increased activity of the methionine cycle observed in these cells, which permits the methylation of specific genes and the activation of multiple survival pathways. It has been recently shown that SET9, a methyltransferase that has a prominent role in the lysine methylation of histones and non-histone proteins, modulates the stability and proapoptotic functions of E2F1.^36^ The methylation of E2F1 has been shown to prevent its acetylation and phosphorylation at distant amino acids, both of which are required for DNA damage-induced stabilization of the protein and the activation of proapoptotic target genes.^36^ These results suggest that inhibiting SET9 in BC cells might be a promising strategy to induce E2F1-dependent apoptosis, preferably in p53-deficient tumors. However, we observed that (R)-PFI-2 was not effective in inducing apoptosis even in irradiated BC cells. Because the promoter methylation of E2F1-dependent proapoptotic genes is a step in apoptotic defense mechanisms,^12^ the simultaneous inhibition of both SET9 and DNMT1 in the presence of DNA damage may be required to favor the tumor-suppressor function of E2F1. Therefore, the therapy proposed in this study could be considered an example of how a global strategy to target the epigenetic machinery of cancer cells may present advantages over therapies that target only specific elements of the complex epigenetic system.

## Materials and Methods

### Reagents and antibodies

3-*O*-(3,4,5-trimethoxybenzoyl)-(−)-catechin (TMCG) was synthesized from catechin via a reaction with 3,4,5-trimethoxybenzoyl chloride.^37^ DIPY and 5-Aza-dC were obtained from Sigma-Aldrich (Madrid, Spain), and (R)-PFI-2 was obtained from Cayman Chemical (Ann Arbor, MI, USA). Antibodies against the following proteins were used: *β*-Actin (Sigma; monoclonal clone AC-15), BRCA1 (Abcam, Cambridge, UK; monoclonal clone MS110), E-Cadherin (Millipore, Madrid, Spain; monoclonal clone 67A4), N-cadherin (Millipore, monoclonal clone EPR1792Y), CD24 (Abcam; monoclonal clone SN3), CD44 (Abcam; monoclonal clone EPR1013Y), Histone H3 dimethyl K79 (Abcam; polyclonal), Histone H4 dimethyl K20 (Abcam; polyclonal), MMSET (Abcam, monoclonal clone 29D1), mono and dimethyl arginine (Abcam; monoclonal clone/E6), phospho-H2AX (Ser139; Millipore; monoclonal clone JBW301), PRMT1 (Abcam; polyclonal), SOX2 (Abcam; monoclonal clone 57CT23.3.4), Vimentin (Millipore, monoclonal clone VIM3B4) and 53BP1 (Abcam; polyclonal).

### Cell cultures and treatments

The MCF7 and MDA-MB-231 human BC cell lines and the murine cell line 4T1 were purchased from the American Type Culture Collection (ATCC), and they were routinely authenticated using genotype profiling according to the ATCC guidelines. Cells were maintained in the appropriate culture medium supplemented with 10% fetal calf serum and antibiotics. Cell viability was evaluated using colorimetric assays to analyze mitochondrial functions and 2,3-Bis(2-methoxy-4-nitro-5-sulfophenyl)-2H-tetrazolium-5-carboxanilide (XTT; Sigma) cell proliferation assays. For these assays, cells were plated in 96-well plates at a density of 1000–2000 cells/well. Unless otherwise indicated, cells were treated with the HMT (consisting of a combination of 10 *μ*M TMCG and 5 *μ*M DIPY) for 72 h. This single concentration dose regimen was chosen based on previous studies carried out in our laboratory, where the effects of single treatments (TMCG or DIPY) on BC cells can also be observed.^12, 19^ For treatments involving HMT combined with IR (IR/HMT), after 3 days of treatment with the HMT, the cells were irradiated at the indicated doses. For ionizing radiation (IR) assays, the cells were irradiated using an Andrex SMART 200E machine (YXLON International, Hamburg, Germany) operating at 200 kV, 4.5 mA with a focus-object distance of 20 cm at room temperature and at a dose rate of 2.5 Gy/min. The radiation doses were monitored using a UNIDOS universal dosimeter in a PTW Farme ionization chamber TW 30010 (PTW-Freiburg, Freiburg, Germany) in a radiation cabin. For fractionated radiation experiments, cells were irradiated using 3 Gy on three consecutive days.

### Mammosphere culture conditions

Mammospheres were cultured in 24-well ultralow-attachment plates (Corning Inc., Lowell, MA, USA). Before plating, primary mammosphere cell suspensions were dissociated by incubating them for 2 min at 37 °C in 0.05% trypsin/EDTA followed by mechanical dissociation using a 22-gauge needle. The cells were then passed through a 40-*μ*m sieve to obtain a single-cell suspension. Single cells were then plated at 500 cells/cm^2^, and secondary mammosphere formation assays were performed in serum-free stem cell medium containing DMEM/F12 (1 : 1) supplemented with B27 (GIBCO/Invitrogen, Barcelona, Spain), 20 ng/ml EGF (Sigma), 10 ng/ml fibroblast growth factor (Sigma) and an antibiotic–antimycotic. Mammosphere-forming efficiency was calculated by dividing the number of mammospheres (colonies >60 *μ*m in diameter) that were formed by the number of viable cells (as determined by trypan blue) at the end of the experiments.^38^ For mammosphere dedifferentiation assays, complete primary mammospheres were cultured in the same culture media used for adherent cells to avoid the extra signaling that can be produced by specific growth factors that are enriched in standard stem cell medium. Each experiment was performed in triplicate.

### Apoptosis assays

Apoptosis was analyzed using an ELISA assay (Cell Death Detection ELISAPLUS, Roche Diagnostics, Barcelona, Spain) to detect mono- and oligonucleosomes in the cytoplasmic fractions of cell lysates using biotinylated antihistone and peroxidase-coupled anti-DNA antibodies. The amount of nucleosomes was photometrically quantified at 405 nm by determining the peroxidase activity that was retained in the immunocomplexes. Apoptosis was defined as the specific enrichment of mono- and oligonucleosomes in the cytoplasm and calculated by dividing the absorbance of the treated samples by the absorbance of the untreated samples after correcting for the number of cells. The induction of apoptosis in each BC cell line after 7 h of treatment with 2 *μ*M staurosporin (100% apoptotic cells) was used to calculate the number of apoptotic cells. The Hoechst staining method was also used to detect apoptosis. Replicate cultures of 1 × 10^5^ cells per well were plated in six-well plates. The cells were subjected to the specified treatments for 72 h. After exchanging the medium for fresh medium, the cells were incubated with 5 *μ*l of Hoechst 33342 solution (Sigma) per well at 37 °C for 10 min and then observed under a fluorescence microscope. Strong fluorescence was observed in the nuclei of apoptotic cells, whereas weak fluorescence was observed in non-apoptotic cells. Quantification of the apoptotic cells was performed by counting the cells in four random fields in each well. Apoptosis in 4T1 cells and 4T1-induced tumors was determined using a DNA ladder assay.^39^ Briefly, 4T1 cells (2 × 10^6^) were homogenized in DMSO (100 *μ*l) and mixed well, followed immediately by vortexing. Equal volume (100 *μ*l) of Tris-EDTA buffer (pH 7.4) with 2% SDS was added, followed by mixing and vortexing. The resulting solution was centrifuged at 12 000 × *g* at 4 °C, and 20 *μ*l of the supernatant was loaded on agarose gels. For the assays with 4T1-induced tumors, frozen tumors (three per treatment) were cut into ~0.1-g slices. Five randomly chosen slices from each tumor were processed as described for 4T1 cells. Densitometry analysis of ladder was carried out using Gel-Pro Analyzer *v*4.0 (Media Cybernetics, Rockville, MD, USA).

### *β*-Galactosidase staining to analyze cellular senescence

The induction of senescence was analyzed by observing morphological changes that were detectable under light microscopy and by analyzing cellular *β*-galactosidase activity, a known marker of senescent cells, using a Senescence Galactosidase Staining Kit (Abcam) according to the manufacturer's protocol. The percentage of *β*-galactosidase-positive cells was counted in 10 random fields at × 20 magnification.

### Migration assays

Cells were seeded at 5 × 10^5^ cells/plate in 60-mm plates and grown in complete medium for 16 h. Subsequently, the cell monolayers were scarred using a sterile micropipette tip and incubated for another 24 h. For each sample, three areas were defined and monitored during this period. Photographs were taken at the beginning of the assay (*t*=0 h) and after 72 h (magnification, × 100).

### Comet assay

DNA damage in cells was evaluated using Single Cell Gel Electrophoresis Alkaline Assays, which were obtained from Trevigen, according to the manufacturer's instructions.

### Cell cycle analysis

BC cells, after the indicated treatments, were resuspended in PBS and fixed in 70% ethanol–PBS for 30 min at 4 °C. Fixed cells were washed with PBS and treated with RNase at 37 °C for 30 min. Finally, the cells were stained with propidium iodide (PI) for 30 min at 37 °C. Samples were analyzed using flow cytometry in a FACSort cytometer (Becton-Dickinson, Franklin Lakes, NJ, USA) and using Cell Quest (BD Biosciences, San Jose, CA, USA) and ModFIT software (Verity Software House, Topsham, ME, USA).

### PCR arrays

Total RNA was isolated using TRIzol reagent (Life Technologies, Barcelona, Spain), and genomic DNA was eliminated from the samples using the DNA-free TM kit (Ambion, Austin, TX, USA). Complementary DNA (cDNA) was produced using an RT2-First Strand Kit (SABiosciences, Frederick, MD, USA). First, an additional genomic DNA elimination reaction was performed and followed by first-strand cDNA synthesis using 1 *μ*g of total RNA. Complementary DNA was subsequently constructed using a Stem Cell PCR array (SABiosciences) according to the manufacturer's directions. Quantitative PCR reactions were performed using the following program in an Applied Biosystems 7900HT system (Applied Biosystems, Foster City, CA, USA): one cycle of 10 min at 95 °C to activate the HotStart DNA polymerase followed by 40 cycles at 95 °C for 15 s and 60 °C for 1 min. The PCR array (PAHS-405Z) contained pre-dispensed primer sets that included 84 stem cell-related genes, five housekeeping genes for normalization to the PCR array data, one genomic DNA contamination control, three reverse transcription controls and three PCR-positive controls. The data were analyzed using the SDS 2.2.2 software and the SABiosciences data analysis tool. Expressions of fold differences were calculated using the -ΔΔ*C*t method. *β*-actin was used as an endogenous control to calculate Δ*C*t values (*C*t_gene of interest_−*C*t*β*_-actin_). Gene expression fold differences were calculated relative to the averaged Δ*C*t value in the control MCF7 cells by determining the ratio of 2^−Δ*C*t^ between the cells of interest and the MCF7 cells (2^−Δ*C*t cell line of interest^/2^−Δ*C*t MCF7 cells^).

### Stealth RNA transfections

Specific Stealth siRNAs for PRMT1 (HSS142549, HSS142550 and HSS142551) were obtained from Life Technologies and transfected into MCF7 cells using Lipofectamine 2000. Treatments were started at 24 h after siRNA transfection. Stealth RNA-negative control duplexes (Life Technologies) were used as control oligonucleotides, and the ability of the Stealth RNA oligonucleotides to knockdown the expression of selected genes was analyzed using western blot analysis at 24 h after siRNA transfection.

### Immunoblotting and immunoprecipitation

Whole-cell lysates were collected by adding SDS to the sample buffer. After extensive sonication, the samples were boiled for 10 min and subjected to SDS-PAGE. The proteins were then transferred to nitrocellulose membranes and analyzed using immunoblotting (ECL Plus, GE Healthcare, Barcelona, Spain). For immunoprecipitation assays, the cells (~5 × 10^6^) were lysed in 500 *μ*l of lysis buffer (50 mM Tris, pH 8.0, 300 mM NaCl, 0.4% NP40, 10 mM MgCl_2_) supplemented with protease and phosphatase inhibitor cocktails (Sigma). The cell extracts were cleared by centrifugation (20 000 × *g* for 15 min) and then diluted with 500 *μ*l of dilution buffer (50 mM Tris, pH 8.0, 0.4% NP40, 2.5 mM CaCl_2_) supplemented with protease and phosphatase inhibitor cocktails and DNase I (Sigma). The extracts were pre-cleared in 30-min incubations with 20 *μ*l of Pure Proteome Protein G Magnetic Beads (Millipore) at 4 °C while being rotated. The antibodies (as indicated in the figure legends) were then added to the pre-cleared extracts. After incubation for 1 h at 4 ºC, 50 *μ*l of Pure Proteome Protein G Magnetic Beads were added, and the extracts were further incubated for 20 min at 4 °C with rotation. After extensive washing, the bound proteins were analyzed using western blots. The unbound extracts were used as the positive inputs to determine protein loading.

### Microscopy

Laser-scanning confocal microscopy of fixed cells was performed using a Leica TCS 4D confocal microscope (Wetzlar, Germany). For indirect immunofluorescence studies, preparations of cells on glass slides were fixed with cold acetone for 5 min and then washed with PBS. The cells were incubated with 3% bovine serum albumin (BSA) for 20 min and then with primary antibodies (diluted 1 : 200 in PBS containing 1% BSA) for 2 h at room temperature. The cells were washed three times in PBS and incubated for 1 h at room temperature with Alexa Fluor Dyes (Life Technologies), which were used as the secondary antibodies. After 3 washes with PBS, the cells were incubated with 0.01% 4'-6-diamidino-2-phenylidene (DAPI; Sigma) in water for 5 min. To determine antibody specificity, primary antibodies were replaced with specific IgGs (diluted 1 : 200) during immunofluorescence. Coverslips were permanently mounted to the slides using fluorescent mounting medium (DAKO, Carpinteria, CA, USA) and allowed to dry overnight in the dark. For confocal microscopy, the mammospheres were fixed in ice-cold methanol and acetone (−20 °C; 1 : 1) for 5 min on ice. The mammospheres were then permeabilized and blocked in 0.1% Triton and 1% BSA in 1 × PBS for 1 h at RT. They were then washed in 1 × PBS for 5 min three times. Primary antibodies against SOX2 and E-cadherin were diluted in 0.2% BSA in 1 × PBS. Mammospheres were incubated in the primary antibodies at 1 : 250 dilutions overnight at 4 °C. The next day, the mammospheres were gently washed in 1 × PBS three times for 5 min at RT. Next, rabbit and mouse conjugated Alexa Fluor secondary antibodies were incubated at a 1 : 1000 dilution at RT for 1 h in the dark with slow agitation. Cells were washed with 1 × PBS three times for 10 min with slow agitation in the dark. Cells were briefly submerged in DAPI to stain them, and they were then washed in distilled, deionized water for 1 min. Coverslips were then permanently mounted to the slides using fluorescent mounting medium (DAKO).

### Mouse BC model

Animals were bred and maintained according to the guidelines of the Spanish legislation on the ‘Protection of Animals used for Experimental and other Scientific Purposes' and in accordance with the directives of the European community. Female BALB/c mice (6 weeks old, 20–24 g) were used. Subconfluent 4T1-*luc2* cells (Caliper Life Sciences, Hopkinton, MA, USA) were harvested and resuspended in PBS at a final density of 1 × 10^7^ cells/ml. Before injection, cells were resuspended in PBS and analyzed using 0.4% trypan blue exclusion assays (viable cells, >90%). For cancer cell injections, ~5 × 10^5^ 4T1-*luc2* cells in 50 *μ*l of PBS were injected into the mammary fat pad of each mouse using a 27-gauge needle. Animals with tumors greater than 8 mm in diameter on day 8 or that showed no visible tumor growth by day 12 were excluded. Groups were subjected to radiotherapy and/or chemotherapy treatments starting on day 15 after the tumor cells were injected. Radiotherapy consisted of a single 5-Gy dose that was delivered with the X-ray irradiator described above. Irradiation was locally confined to the tumors by shielding the rest of the body of the mouse with lead. Radiotherapy was performed on days 16, 18, 20, 24, 26 and 28 (a total of 30 Gy/mice) after tumor injection, whereas chemotherapy (HMT) was administered intraperitoneally on days 15, 17, 19, 21, 23, 25 and 27 and consisted of a mixture of TMCG (30 mg/kg/day) and DIPY (10 mg/kg/day). Control mice received the same volume of vehicle (DMSO). Primary tumors and metastases were analyzed on day 40 using an IVIS Imaging System (Caliper Life Sciences). Animal procedures were approved by the Ethical Committee of the University of Murcia and the Direccion General de Ganaderia y Pesca, Comunidad Autonoma de Murcia (Project reference A1320140710).

### Image acquisition, quantification of western blots and statistical analysis

Western blot analyses and analyses of microscopy data were repeated at least three times, with similar results. The results from one experiment are shown. To quantify the results, the western blots were scanned using a Bio-Rad ChemiDoc scanning densitometer (Bio-Rad Laboratories, Hercules, CA, USA). For other experiments, the mean±S.D. of three determinations performed in triplicate were calculated. Numerical data were analyzed to determine statistical significance using Mann–Whitney tests for comparisons of means in the SPPS statistical software for Microsoft Windows, release 6.0 (Professional Statistic, Chicago, IL, USA). Individual comparisons were made using Student's two-tailed, unpaired *t*-tests. The criterion for significance was *P*<0.05 for all comparisons.

## Abbreviations

BC: breast cancer
CIC: cancer-initiating cell
DBR: double-strand break
DDR: DNA damage response
DNMT1: DNA methyltransferase 1
DIPY: dipyridamole
EMT: epithelial–mesenchymal transition
ER: estrogen receptor
Gy: Gray
HMT: hypomethylating treatment
IR: ionizing radiation
MFE: mammosphere-forming efficiency
NLS: nuclear localization signal
PARP: poly-ADP-ribose polymerase
PR: progesterone receptor
PRMT1: protein arginine methyltransferase 1
SAH: S-adenosylhomocysteine
SAM: S-adenosylmethionine
TMCG: 3-*O*-(3,4,5-trimethoxybenzoyl)-(-)-catechin
