# Supplementary material for: Targeting the epigenetics of the DNA damage response in breast cancer
Source: Cell Death Dis. 2016 Apr 7;7(4):e2180–. doi: 10.1038/cddis.2016.85 (PMC4855664; doi:10.1038/cddis.2016.85)
Supplement: Supplementary Information [file cddis201685x1.pdf]

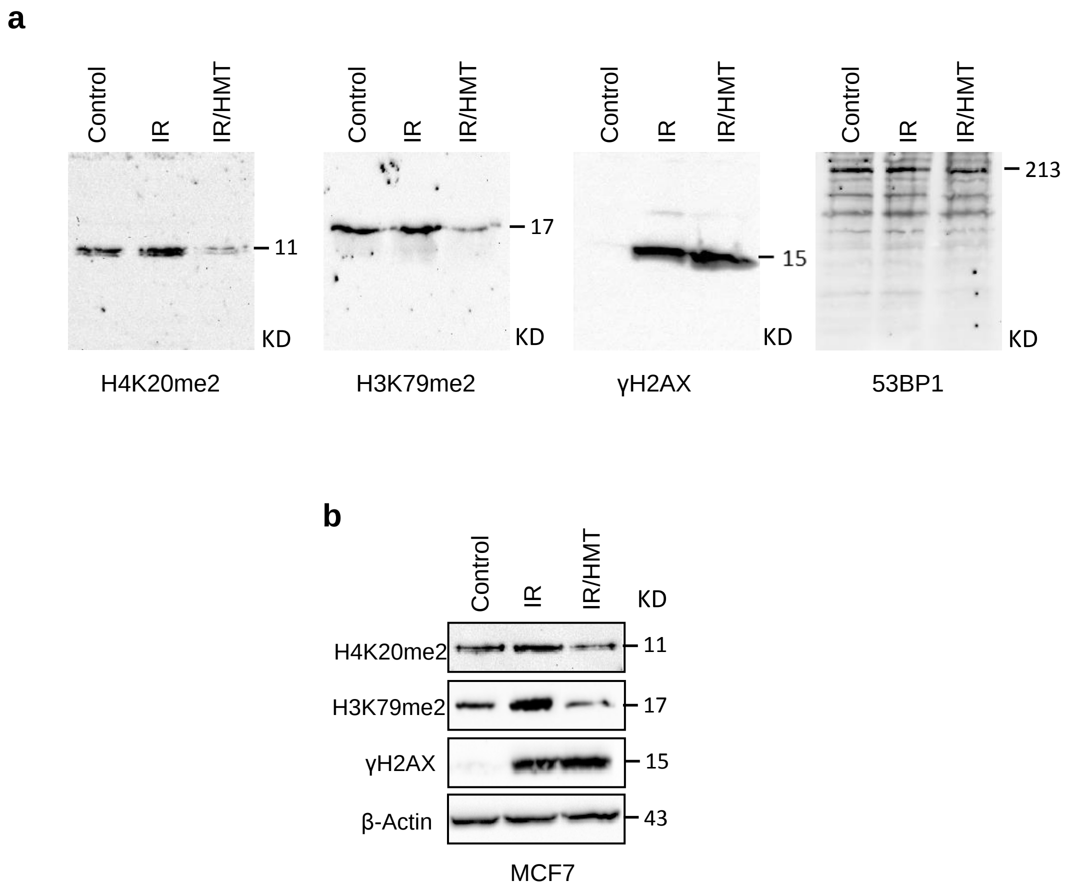

Supplementary Figure 1. (**a**) Uncropped western blots corresponding to Figure 2a to show the specificity of the employed antibodies. (**b**) The total levels of H3K79me2, H4K20me2, and γH2AX were examined in MCF7 cells using western blot analysis following the indicated treatments. At 1 h after irradiation (120 Gy), the cells were collected and analyzed.
